# Unusual Skull Metastasis From Thyroid Cancer: A Case Report

**DOI:** 10.7759/cureus.77234

**Published:** 2025-01-10

**Authors:** Julieth A Guzman Lopez, Julio C Díaz Acosta, Oscar E Rivera Contreras, Carlos J Zamora Rangel, Edgar F Manrique Hernández

**Affiliations:** 1 Internal Medicine, Universidad Industrial de Santander, Bucaramanga, COL; 2 Head and Neck Surgery, Unidad Especializada de Cabeza, Cuello y Tórax de Santander, Bucaramanga, COL; 3 Internal Medicine, Universidad de Antioquia, Medellín, COL; 4 Pathology, Unidad Especializada de Cabeza, Cuello y Tórax de Santander, Bucaramanga, COL; 5 Epidemiology, Fundación Cardiovascular de Colombia, Floridablanca, COL

**Keywords:** diagnosis, metastasis, skull, thyroid cancer, unknown primary tumor

## Abstract

Papillary thyroid carcinoma is a common condition, but skull metastases are extremely rare. This case involves an older adult who presented with a progressively growing mass in the skull that also involved the orbit. Imaging revealed a lesion measuring 85x76x91 mm, causing compression of the left frontal lobe and midline deviation. The patient underwent craniotomy with tumor resection and orbital exenteration, which led to the diagnosis of metastasis from papillary thyroid carcinoma. A total thyroidectomy was later performed, but histopathology found no evidence of malignancy in the thyroid gland.

Skull metastasis in patients with thyroid cancer is rarely reported, and its diagnosis, as well as pre-and postoperative management, remains uncertain. Additionally, the occurrence of metastasis without identifying the primary tumor in the thyroid gland is an uncommon phenomenon. Only two previous cases have been reported where skull metastasis occurred without a detectable primary thyroid tumor. Skull metastases are rare, and diagnosing and treating them presents significant challenges. However, a skull metastasis originating from the thyroid, even in the absence of a primary tumor, is a rare but real possibility.

## Introduction

Differentiated thyroid carcinoma (DTC) is a prevalent pathology, with incidence increasing by more than 300% over the past three decades. It accounts for 2% of all invasive cancers, and its presentation is three times greater in women than in men. The papillary subtype is the most frequent, which accounts for roughly 90% of cases [1].

Various studies have described the presence of distant metastasis, with a frequency of 1 to 23% [2]. The lungs are the most common site, followed by bone, with frequencies of 53% and 28.1%, respectively [3]. Bone metastases are usually found in the axial skeleton, ribs, pelvis, long bones, and sternum [4], while they are infrequently found in the skull and are extremely rare in the frontal area [5]. Therefore, after authorization by informed consent, we have described the clinical, imaging, and histopathological findings of the case of a patient with skull metastasis of papillary thyroid cancer without identification of the primary tumor.

## Case presentation

An 88-year-old female patient from Northeastern Colombia with a history of hypertension and type 2 diabetes mellitus with adequate management and metabolic control sought a medical consult for progressive growth of an indurated mass in the left lateral frontal area, with proptosis and loss of ipsilateral vision, and a two-year progression.

**Figure 1:**
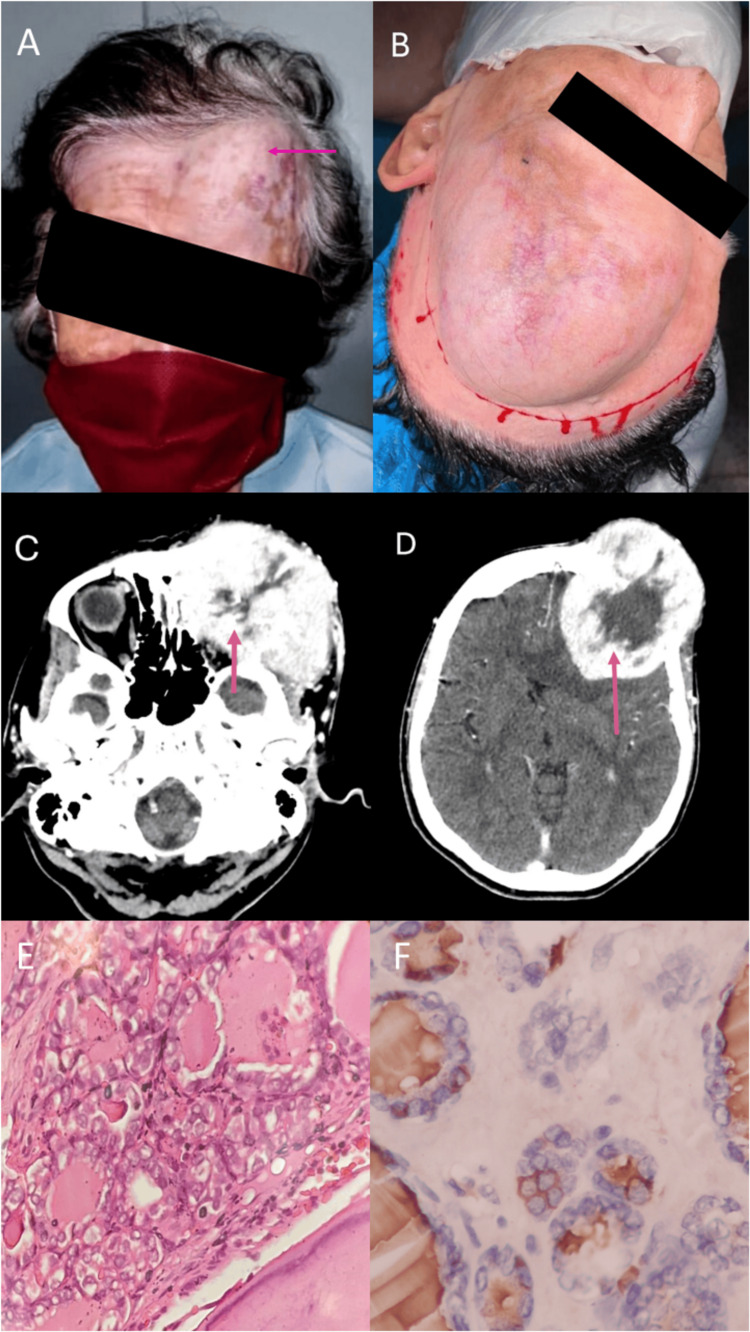
Clinical, radiological, and histopathological findings of a large malignant thyroid tumor with skull infiltration and intracranial extension A and B: Depiction of a patient with a 15x10 cm mass on the left fronto-orbital region showing apparent ipsilateral ocular involvement. C:  Contrast CT demonstrates a large tumor mass measuring 85x76.6x91 mm, infiltrating the bone tables of the left fronto-orbital skull and the major wing of the sphenoid. D: Contrast CT shows a solid, hypervascular, infiltrative lesion involving the calvarium in the left frontal region, causing compressive effect on the ipsilateral frontal parenchyma, with slight midline deviation and collapse of the frontal horn of the lateral ventricle. Significant thick peripheral enhancement is observed after administering the contrast agent. E: Illustrates a malignant epithelial tumor originating from the thyroid, with oval nuclei cells and intranuclear inclusions infiltrating the bone tissue of the skull (40x microscopy, H&E). F: Shows antigenic expression of thyroglobulin (40x microscopy, Immunohistochemistry).

On physical examination, the patient was afebrile, hemodynamically stable, and had anthropometric measurements within normal ranges. The head-neck area showed evidence of an enlarged left frontal, temporal region from an indurated mass measuring approximately 10x10 cm, with an inability to open and move the eyes. In the thyroid region, the enlarged left lobe was palpated, with no evident signs of focalization or neurological deficit (Figure 1a-1b).

A thyroid ultrasound was performed, which reported a multinodular goiter with hyperechoic, solid, circumscribed, and multifocal nodules and a larger nodule in the left lobe measuring 13x8.2x7.6mm, with no suspicious lymph nodes. Thyroglobulin is 3,147ng/ml, antithyroglobulin antibodies 42 ng/ml, and TSH 0.39 ng/ml. It was decided to perform a total thyroidectomy with central emptying, which resulted in a negative pathology report for malignancy.

The CAT report of the skull showed the presence of a lesion with a neoplastic appearance, with well-defined contours measuring 85x76.6x91 mm (a previous study from one year earlier showed a lesion measuring 65x65x60 mm), centered in the left frontal bone with compressive effect on the left frontal parenchyma, infiltrative involvement of the orbital roof and likely extending to the ipsilateral dural (Figure 1c-1d). In light of these findings, it was decided to perform a surgical approach in conjunction with neurosurgery, craniotomy for supratentorial tumor resection with complete orbital excision and reconstruction with neurovascular island flap. A giant protruding lesion was identified, measuring approximately 20x20 cm, completely destroying the skull, invading the epidural, subdural, and dura mater regions, with significant ipsilateral compression of the temporal lobe. The patient tolerated the procedure without complications. The histopathological report showed a metastatic malignant tumor originating from the thyroid with compromised bone margins. The immunohistochemistry was positive for AE1/AE3, TTF 1, CK 19, thyroglobulin and CK 7 markers. The Ki 67 proliferation index was 10%. Findings confirmed follicular variant of papillary thyroid carcinoma (Figure 1e-1f). During follow-up, the patient died secondary to decompensation of underlying cardiovascular diseases, independent of the oncologic pathology.

## Discussion

DTC metastasis to the bone is the second most common metastasis after the lungs. However, its finding in the skull has been reported in 2.5-5.8%, the most common sites being the occiput and the base of the skull. The follicular subtype is mainly predominant, followed by papillary [5]. There have been 11 published cases of papillary thyroid cancer metastasis to the frontal skull (Table 1) [6-16], most of which have been in older adults, with a mean age of 64 years, and mostly women. Nearly all cases had a previous or concomitant diagnosis of papillary thyroid cancer, except for the present case and one reported by Begum et al. [6], where the primary tumor could not be identified.

**Table 1:** Characteristics of case reports of frontal skull metastasis of papillary thyroid carcinoma * Near total thyroidectomy for thyroid adenoma; ** Metastasis to humerus;  *** Died before receiving any treatment NM: No mention; LTL: Left thyroid lobe; F: Female; M: Male; RAI: Radioactive iodine; TT: Total thyroidectomy; TR: Tumor resection

Year, Author	Age/Sex	Symptomatology	Thyroidectomy	Primary Thyroid	Treatment	Subtype	Survival
2006. Hashiba et al. [10]	74/F	Painful mass	23 years before	NM	TR, RAI	Papillary	Alive after 3.5 years
2009. Feng et al. [11]	62/F	Headache, Painful mass	NM	NM	TT, TR	Papillary	Alive after 6 months
2011. Houra K et al. [9]	76/F	Headache, confusion, Sensation of a mass, changes in sleep	13 years before	NM	TR	Papillary	Alive after 10 months
2013. Li et al. [16]	61/F	Sensation of a mass	13 years before*	1.4cm LTL	TR, TT and RAI	Papillary	Alive after 2 years
2014. Mendes et al. [12]	29/F	Sensation of a mass	Concomitant	NM	TT	Follicular Papillary variant	NM
2015. Begum et al. [6]	55/F	Painful mass	8 months antes	Not identified	Radiotherapy and RT	Papillary	NM
2017. Pyo JY [13]	25/M	Asymptomatic	7 years before left hemithyroidectomy	Both lobes	TR, RAI and Radiotherapy	Follicular Papillary variant	Alive after 15 years
2018. Sheikh et al. [15]	54/M	Painful mass	Concomitant	10x6.5x6 cm LTL**	TR, TT, and Radiotherapy	Follicular Papillary variant	NM
2018. Barwad et al. [14]	51/M	Sensation of a mass	Concomitant	15x13x12cm diffuse	Not received***	Papillary	Death
2021. Moen et al. [7]	75/F	Sensation of a mass	Concomitant	Istmo 3.65cm	TT, RAI, and Radiotherapy	Papillary	NM
2022. Sirko et al. [8]	55/M	Painful mass, loss of movement in contralateral extremities	After tumor outcome	NM	TR, TT, RAI, and Radiotherapy	Follicular Papillary variant	Alive after 1 year
2024. Guzmán (own)	88/F	Sensation of a mass and loss of sight	Concomitant	Not identified	TT, TR	Follicular Papillary variant	Death

In most patients, DTC presents as a localized pathology with an excellent prognosis and a 10-year survival rate of 80-95% [3]. However, survival can drop with the presence of distant metastases, with a very wide range of 38-75% at 10 years [17]. Survival of bone metastases is between 13-23% at 10 years [18]. An average survival of 3.1 to 4.5 years has been reported for patients with metastases of the skull [5]. In the published cases, most of the patients are described as alive during follow-up, with the exception of the case reported by Barwad et al. [14] and the present case. In the former, the patient died 4 months after the metastasis was identified without receiving any type of treatment, and in ours, the patient died after surgery from secondary causes due to other underlying diseases. Although there is little information about the role of comorbidities in metastatic thyroid cancer, the presence of comorbidities has been reported to be key to therapeutic decisions for patients with thyroid cancer, with a large difference observed between the prognosis of patients without comorbidities and those with one or two or more diseases, these latter ones having survival rates of 87%, 61% and 45% at five years, respectively [19].

DTC metastases of the skull are difficult to diagnose due to the unusual nature of their presentation. Differential tumors such as meningioma, chondrosarcoma, and osteoma could be suspected as a first step [7,11]. The most notable symptomatology in most of the published cases is the sensation of progressive growth of a painless mass over five months to four years. The presence of neurological compromise and other associated alterations are infrequent findings, having been reported in a few cases [8,9]. The approach to identifying these tumors has been based on diagnostic suspicion with the use of images such as computerized axial tomography or nuclear magnetic resonance imaging, with which infiltrative lesions with osteolytic characteristics can be found, which usually have a prominent vascular component [5]. For patients with DTC and skull metastasis, the period from initial diagnosis to detection of metastasis has been found to be an average of 23.3 years. However, detection was concomitant in five of the published cases, including ours [17,7,11].

It is difficult to find cases in which primary carcinoma in the thyroid is not identified, but it occurs. There are few reported cases, and the mechanisms involved are not known [20]. Nevertheless, different hypotheses have emerged that could explain this rare phenomenon, such as the non-identification of microcarcinomas < 3mm despite an exhaustive pathological examination and the spontaneous regression of the tumor, as described for other types of cancer, such as melanoma. Fibrosis is the most commonly related histopathological characteristic [20]. The present case is the second report of skull metastasis without identification of a primary tumor, after one published by Begun et al. in which 8 months after total thyroidectomy for a multinodular goiter, a patient was diagnosed with skull metastasis after the progressive growth of a mass in the frontal region [6].

At the moment, there are no clinical practice guidelines for the clear management of these patients. Total thyroidectomy is recognized as the standard treatment for cases of metastatic thyroid cancer, with subsequent individualization of other approaches such as radioactive iodine, local excision of the tumor, radiotherapy, and even bone resorption inhibitors such as bisphosphonates and denosumab, which are still under study for this type of tumor. It is notably important to continue research in the field of conventional and novel therapies to achieve increasingly more adequate treatment [4].

## Conclusions

The presence of distant metastasis in patients with thyroid cancer, especially in areas as uncommon as the skull, poses a significant challenge for diagnosis and treatment. Skull metastases are rare and often hard to identify, which can delay the correct diagnosis. Early suspicion, combined with timely imaging and pathology results, plays a crucial role in detecting and managing these cases effectively. In this case, despite the absence of a clear primary tumor in the thyroid gland, a comprehensive diagnostic approach allowed for the identification of the metastasis, although the patient's underlying conditions complicated the outcome.

Given the limited guidelines on managing thyroid cancer with skull metastasis, treatment decisions must be tailored to each individual case. Surgery, along with adjunctive therapies such as radioactive iodine and medications like bisphosphonates and denosumab, remains the standard. However, more research is necessary to develop clear protocols for this rare presentation. Continuing to study these unusual cases will be key to improving both the treatment strategies and the long-term prognosis for patients facing similar conditions.
